# The role of neuropsychiatry in the care of children and adults with cerebral palsy

**DOI:** 10.1192/bjo.2022.62

**Published:** 2022-06-01

**Authors:** Aaron J. Hauptman, Elizabeth Barkoudah

**Affiliations:** Department of Psychiatry, Kennedy Krieger Institute, Maryland, USA; and Department of Psychiatry, Johns Hopkins University School of Medicine, Maryland, USA; Department of Neurology, Boston Children's Hospital, Harvard Medical School, Massachusetts, USA

**Keywords:** Cerebral palsy, neuropsychiatry, psychiatry, neurodevelopmental disability, multidisciplinary care

## Abstract

Neuropsychiatric symptoms are commonly reported in cerebral palsy. These symptoms interact in complex ways with the core motoric features of cerebral palsy, and require specialised care. We argue for increased awareness of these symptoms by clinicians, and the need for greater integration of neuropsychiatric specialists into the core teams involved in multidisciplinary care for individuals with cerebral palsy and their families.

‘The neuropsychiatrist is … ideally suited to evaluate and treat patients who have abnormalities in perception, cognition, emotion, and/or behavior due to a known psychiatric or neurologic disorder; due to the simultaneous presence of, or interaction between, psychiatric and neurologic disorders (or their treatments), and associated psychosocial elements; or due to an unknown underlying brain condition.’^1^

Neuropsychiatrists are ideally situated to be part of multidisciplinary care teams treating children and adults with cerebral palsy, as well as other acquired and developmental brain conditions. Although accessing routine mental healthcare for individuals with cerebral palsy already poses a significant challenge, we wish to emphasise that the neuropsychiatrist is uniquely well-situated to play a role in team-based care, given the challenges of overlapping neurological and psychiatric symptoms, complex polypharmacy and psychosocial circumstances facing these individuals and their families.

Neuropsychiatrists specialise in relationships between brain injury and symptoms within the domains of cognition, emotion and behaviour. Clinicians in this field are trained either through independent residencies in psychiatry and neurology, or as part of an integrated fellowship following a single residency in either field.^1^ Some clinicians also have paediatric subspecialty training.

Neuropsychiatrists often work in multidisciplinary teams in the coordinated care of individuals with complex neurological and psychiatric conditions by assessing if a given symptom is the result, for example, of an underlying neurological insult, a primary psychiatric condition, iatrogenic factors or some other cause. They then assist with the provision of psychoeducation; collaborate with colleagues in other core specialties such as neurology, physiatry, gastroenterology, orthopaedics and neurosurgery; and help manage psychiatric and neurological medications. This can help maximise treatment benefits and minimise risks.

Juxtaposed with other medical and mental health providers, neuropsychiatrists focus on neuroanatomical correlates of behavioural symptoms in brain-based disorders, as well as having training in a range of psychotherapies and pharmacological management, and play a role in translating between different specialties that may focus more specifically on features of either mental or physical illnesses. As such, this places them in a unique position in the care of individuals with cerebral palsy.

Cerebral palsy is a heterogeneous condition that involves permanent motor dysfunction owing to a nonprogressive neurodevelopmental or acquired injury during the pre-, peri- or early postnatal period.^2^ Causes can be multifactorial, including neurogenetic causes, injury to the developing brain (i.e. perinatal stroke, hypoxia, infection) and postnatal brain injury, among others, with prematurity being the most common association. Although the definitions commonly used do not include other neurological, behavioural and emotional sequelae, and comorbidities, they are often acknowledged in definitions as symptoms commonly accompanying the core motoric features.^3–5^

Mental health concerns are widely acknowledged across studies within populations of individuals with cerebral palsy, and are thought to affect 50% of individuals or more, by some reports; however, data are limited, utilise heterogeneous measures and offer limited insight into the relationship between mental health outcomes, motoric functional levels and neurocognitive symptoms of cerebral palsy.^6^ Beyond mental health diagnoses considered as comorbidities, the complex interrelationship between neurological and psychiatric components of cerebral palsy are infrequently discussed, but can often be lynchpins in the management of an individual's symptoms. At the same time, it is sometime easier to overlook this interrelationship, or assume that any symptom experienced by individuals with cerebral palsy are caused by their cerebral palsy. This link is not necessarily related to the severity of the motor disability, but may be tied to factors such as pain, stress levels, coping skills or social supports.^7^

Adults with cerebral palsy have markedly elevated rates of polypharmacy and hyper-polypharmacy compared with adults without cerebral palsy, and children with severe neurological impairment who have more high-distress symptoms have an associated higher risk of polypharmacy.^8,9^ Given the increased risk of side-effects, the potential for medications being used for one purpose (e.g. focal epilepsy) resulting in side-effects within a different domain (e.g. irritability) and the frequent challenges in identifying the causal relation between these, there is a role for clinicians specialising in these interrelationships and for close coordination between psychiatric and neurological providers.

An example of the challenging relationship between a motor phenomenon common in cerebral palsy and psychiatric symptoms occurs in the case of spasticity, the velocity-dependent muscular tone increase with involuntary contraction of muscles seen in individuals with upper motor neuron injuries. Spasticity occurs in about three-quarters of individuals with cerebral palsy.^9^ Although it remains underresearched, we have seen clinical instances where the bidirectional relationship between these motor symptoms and a patient's psychiatric symptoms was at the core of their suffering. For instance, we have co-managed patients where management of comorbid psychiatric symptoms, such as depression, anxiety and insomnia, improved spasticity; similarly, we have seen cases where successful management of spasticity (e.g. with intrathecal baclofen) has alleviated psychiatric symptoms. There are hints in the literature to suggest this bidirectional relationship, such as findings that interventions targeting spasticity (e.g. by using botulinum-A toxin injections) have been shown to improve sleep quality in children with cerebral palsy.^10^ There is evidence from other upper motor conditions, too, such as findings that show worsening of perception of spasticity with stress and anxiety in multiple sclerosis and stroke.^11^ As a result of these complex interrelationships, good psychiatric management affects the status of core symptoms of cerebral palsy.

Many other complex neuropsychiatric comorbidities can be seen in cerebral palsy. For example, many individuals may carry additional diagnoses, such as mood disorders, autism spectrum disorder and epilepsy.^12^ Each of these may require their own complex treatments. Taking epilepsy as a common example, a frequent side-effect of some anti-epileptic agents is agitation and irritability.^13^ The question may arise as to why an individual with cerebral palsy is experiencing agitation and irritability. This could be caused by anti-epileptic medication side-effects, but could also be a result of mood or anxiety disorders, pain, sedation, undertreatment of seizures or psychosocial stress. Although the neurologist will manage these core neurological conditions and a general psychiatrist, social worker or psychologist would be comfortable with diagnosis and management of most psychiatric comorbidities, the neurologically informed exploration of these behavioural concerns falls ideally in the realm of neuropsychiatry. Helping to arrive at a treatment regimen maximising efficacy of the fewest possible medications (say, by increasing an anti-epileptic agent to cover partially treated, nocturnal seizures, thus improving daytime fatigue and irritability) is clearly in the patient's best interest.

Finally, we would like to highlight an example of the impact of neuropsychiatric psychoeducation. We recall an individual with severe aggressive behaviours in the setting of perinatal intraventricular haemorrhage with ventriculoperitoneal shunt and right-sided encephalomalacia with resultant left-sided spastic hemiplegia. The patient's aggression often stemmed from deficits in social pragmatics and impulsivity, with limited ability to self-regulate when upset or anxious. The patient's aggression could be destructive and dangerous, and the family reported feeling unsafe at home. They felt at a loss as to why, as their child became a teenager, baseline disinhibition had developed into such overt aggression. Before neuropsychiatric consultation, the family had a strong understanding of the motor and cognitive effects of cerebral palsy, as explained by their clinical team in the first few years of their child's life. However, they did not feel that they had an understanding of the neurobehavioural effects of their child's injury. The family described that appreciating the specifics of their child's brain injury, data about neuropsychiatric comorbidities in cerebral palsy and possible neuroanatomical correlations between involved brain regions and psychiatric symptoms was particularly helpful. This opened doors to specific behavioural and pharmacological interventions, resulting in dramatic improvements.

One challenge is that there is a dearth of neuropsychiatrists. There are currently 473 diplomates in the subspecialty of behavioural neurology and neuropsychiatry listed on the website of the United Council for Neurologic Subspecialties, the board that provides the subspecialty's accreditation.^14^ This is a small number, given the need for clinicians trained at this interface. Furthermore, there are an even smaller number of clinicians doing such work in the paediatric realm. Numerous reasons likely exist for this limited number of clinicians. Training is longer than in general psychiatry or neurology, lasting up to 6 or more years; clinics and hospitals often may not provide the additional scaffolding, administrative or financial supports often necessary for such complex care; siloing of hospital systems often results in challenges with creating multidisciplinary clinics (already affected by limited provider numbers in other fields essential to cerebral palsy care); and, anecdotally, high rates of burnout exist in complex care specialties.

We would like to see a model where a core multidisciplinary team, including a neuropsychiatrist, shares the care of an individual. This would mean either serial appointments with core team members such as a neurologist with specialisation in developmental disabilities, a physiatrist, a neuropsychologist, a neuropsychiatrist and other providers; or perhaps even appointments with providers of these different disciplines present together at the same time, to maximise communication. This is juxtaposed with the model where neuropsychiatry is consulted externally, often later in treatment and only in more extreme cases. If the neuropsychiatrist were involved in the diagnostic and initial planning phases, and not just treatment-refractory ones, a broader understanding of the individual's needs, and perhaps more rapid resolution of neurobehavioural concerns, could be reached.

## Data Availability

Data availability is not applicable to this article as no new data were created or analysed in this study.
